# Supplementary material for: Knowledge, attitude and practices of pediatricians regarding the prevention of oral diseases in Italy
Source: BMC Public Health. 2006 Jul 5;6:176. doi: 10.1186/1471-2458-6-176 (PMC1543635; doi:10.1186/1471-2458-6-176)
Supplement: Additional file 1 — QuestionnaireBMC : Questionnaire used in the survey [file 1471-2458-6-176-S1.doc]

KNOWLEDGE, ATTITUDE AND PRACTICES OF PEDIATRICIANS REGARDING

THE PREVENTION OF ORAL DISEASES

## DEMOGRAPHIC CHARACTERISTICS

**A1.** How old were you on your last birthday_________(years) **A2.** What is your sex? M ٱ F ٱ

**A3.** In which city is your principle practice setting located?_____________ **A4.** Number of years since graduation_________

**A5.** What is your main practice setting and how long have you been in practice (years)____________________________

**A6.** How many hours do you work in the typical week? ٱ<10 ٱ 11-20 ٱ 21-30 ٱ 31-40 ٱ>40

**A7.** How many patients do you visit in the typical workday?________

### KNOWLEDGE

**B1.** Which of the following is risk factor for oral diseases?

Dental caries Gingivitis Malocclusions

RISK FACTORS

|  | **Yes No Do not know** | **Yes No Do not know** | **Yes No Do not know** |
| --- | --- | --- | --- |
| Gender | ٱ ٱ ٱ | ٱ ٱ ٱ | ٱ ٱ ٱ |
| Anatomy of oral cavity | ٱ ٱ ٱ | ٱ ٱ ٱ | ٱ ٱ ٱ |
| Family tendency | ٱ ٱ ٱ | ٱ ٱ ٱ | ٱ ٱ ٱ |
| Frequency intake of sugar | ٱ ٱ ٱ | ٱ ٱ ٱ | ٱ ٱ ٱ |
| Bottle feeding | ٱ ٱ ٱ | ٱ ٱ ٱ | ٱ ٱ ٱ |
| Breast feeding | ٱ ٱ ٱ | ٱ ٱ ٱ | ٱ ٱ ٱ |
| Nonnutritive sucking habits | ٱ ٱ ٱ | ٱ ٱ ٱ | ٱ ٱ ٱ |
| Poor oral hygiene | ٱ ٱ ٱ | ٱ ٱ ٱ | ٱ ٱ ٱ |
| Inadequate teeth brushing | ٱ ٱ ٱ | ٱ ٱ ٱ | ٱ ٱ ٱ |
| Malpositioned teeth | ٱ ٱ ٱ | ٱ ٱ ٱ | ٱ ٱ ٱ |

###### ATTITUDE

#### Indicate your attitude with each of the following statements.

Agree Uncertain Disagree

**C1.** Dental caries may be prevented ٱ ٱ ٱ

**C2.** Oral hygiene is important in preventing dental caries ٱ ٱ ٱ

**C3.** Fluoride supplement is important in preventing dental caries ٱ ٱ ٱ

**C4.** Pediatrician should provide an oral cavity health examination ٱ ٱ ٱ

**C5.** Routine dental visit is important in preventing oral diseases ٱ ٱ ٱ

**C6.** Gingivitis may be prevented ٱ ٱ ٱ

**C7.** Malocclusion may be prevented ٱ ٱ ٱ

**C8.** Pediatrician has an important role in the prevention of oral diseases ٱ ٱ ٱ

###### BEHAVIORS

**D1.** Do you perform a dietary habits assessment of your patients? **ٱ** Yes **ٱ** No

**D2.** Do you perform an oral health examination of your patients?

**ٱ** No (**go to D3**)

**ٱ** At all (specify the frequency) ____________

**ٱ** Only on mother request

**ٱ** Only patients in presence of a problem

**D3.** At what age do you perform the first oral health examination? ____________

**D4.** At what age do you recommend the first oral health examination? _______ **ٱ** Do not recommend

**D5.** With which frequency do you recommend the oral health examination?

**ٱ** Once a year **ٱ** Every 6 months **ٱ** Every 4 months **ٱ** Every month **ٱ** Only in presence of a problem

**D6.** Do you prescribe dietary fluoride supplementation at your patients?

**ٱ** No (give the reason) _________________________________________________________(**go to D11**)

ٱ At all Starting age_____ Ending age______

ٱ Only on mother request

**D7.** Do you ask at the parents what kind of water the baby drink? ٱ Yes ٱ No

**D8.** Do you modify fluoride prescription in relation to fluoride level in the community drinking water? ٱ Yes ٱ No

**D9.** Indicate the daily dosage of fluoride supplementation you prescribe to your patients who drink water with low fluoride concentration.

|  | **0,10 mg** | **0,15 mg** | **0,25 mg** | **0,30 mg** | **0,40 mg** | **0,50 mg** | **0,70 mg** | **1,00 mg** | **1,50 mg** |
| --- | --- | --- | --- | --- | --- | --- | --- | --- | --- |
| **0-2 years** |  |  |  |  |  |  |  |  |  |
| **2-4 years** |  |  |  |  |  |  |  |  |  |
| **4-6 years** |  |  |  |  |  |  |  |  |  |
| **> 6 years** |  |  |  |  |  |  |  |  |  |

**D10.** Do you advise the parents about the use of fluoridated toothpaste? ٱ Yes ٱ No

**D11** Do you advise the parents about topical application of fluoride? ٱ Yes ٱ No

D12. Do you advise the parents about other way to fluoride somministration? ٱ Yes ٱ No

**D13.** Do you recommend to the parents to wash their children’s teeth? ٱ Yes ٱ No

D14. Do you advise other way to prevent oral diseases? ٱ Yes ٱ No

**D15.** Do you inform the parents about the importance of oral hygiene? ٱ Yes ٱ No

**D16**. Do you provide educational materials to parents about interventions for the prevention and control of oral diseases?

ٱ Never ٱ Rarely ٱ Sometimes ٱ Often ٱ Always

E. INFORMATION

**E1.** Where do you get information about the prevention of oral diseases?

ٱ None ٱ Associations ٱ Scientific journal ٱ Colleagues ٱ Continuing educational courses

ٱ Other (please specify) _______________

E2. Do you think you need further information about the prevention of oral diseases? ٱ Yes ٱ No
